# Thioalbamide, A Thioamidated Peptide from *Amycolatopsis alba*, Affects Tumor Growth and Stemness by Inducing Metabolic Dysfunction and Oxidative Stress

**DOI:** 10.3390/cells8111408

**Published:** 2019-11-08

**Authors:** Luca Frattaruolo, Marco Fiorillo, Matteo Brindisi, Rosita Curcio, Vincenza Dolce, Rodney Lacret, Andrew W. Truman, Federica Sotgia, Michael P. Lisanti, Anna Rita Cappello

**Affiliations:** 1 Department of Pharmacy, Health and Nutritional Sciences, University of Calabria, Via P. Bucci, 87036 Rende (CS), Italy; f.luca90@hotmail.it (L.F.); fiorillo.marco86@gmail.com (M.F.); matteo_brindisi@libero.it (M.B.); rosita.curcio@unical.it (R.C.); vincenza.dolce@unical.it (V.D.); 2 Translational Medicine, School of Environment and Life Sciences, Biomedical Research Centre (BRC), University of Salford, Greater Manchester M5 4WT, UK; 3 Department of Molecular Microbiology, John Innes Centre, Colney Lane, Norwich NR4 7UH, UK; rlacret@yahoo.com (R.L.); andrew.truman@jic.ac.uk (A.W.T.)

**Keywords:** thioviridamide-like molecules, RiPPs, natural products, anticancer agent, cancer stem cells

## Abstract

Thioalbamide, a thioamidated peptide biosynthesized by *Amycolatopsis alba*, is a thioviridamide-like molecule, and is part of a family of natural products representing a focus of biotechnological and pharmaceutical research in recent years due to their potent anti-proliferative and cytotoxic activities on malignant cells. Despite the high antitumor potential observed at nanomolar concentrations, the mechanisms underlying thioalbamide activity are still not known. In this work, the cellular effects induced by thioalbamide treatment on breast cancer cell lines were evaluated for the first time, highlighting the ability of this microbial natural peptide to induce mitochondrial dysfunction, oxidative stress, and apoptotic cell death. Furthermore, we demonstrate that thioalbamide can inhibit the propagation of cancer stem-like cells, which are strongly dependent on mitochondrial function and are responsible for chemotherapy resistance, metastasis, and tumor recurrence.

## 1. Introduction

**Figure 1:**
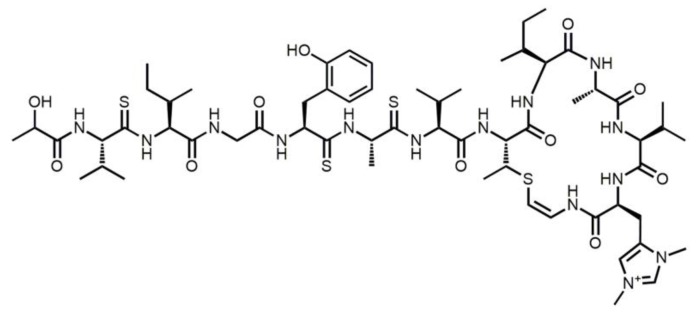
Thioalbamide structure.

Thioalbamide (Figure 1), a recently identified natural product [1], is biosynthesized by *Amycolatopsis alba* DSM 44262, and belongs to the family of ribosomally synthesized and post-translationally modified peptides (RiPPs). This class of natural products is generated by a series of post-translational modifications occurring to a precursor peptide by the action of pathway-specific enzymes, which are encoded in a gene cluster [2]. Precursor peptides can undergo a wide range of post-translational modifications, resulting in mature RiPPs with peculiar chemical structures and biological activities, which spark wide interest from the pharmaceutical point of view. In particular, thioalbamide belongs to the thioviridamide-like molecules (TLMs), a family of compounds recently identified thanks to genome mining studies [1,3] following the initial activity-guided discovery of thioviridamide [4]. The chemical peculiarity of this class of molecules is the presence of different thioamide groups in the peptide backbone, a feature rarely found in natural products [5,6].

Recent studies have shown that TLMs display antiproliferative and cytotoxic effects in different tumor cell lines [7,8], and in particular, thioalbamide has been shown to have activity at nanomolar concentrations against breast, pancreatic, alveolar, and cervical cancer cells [1]. Interestingly, thioalbamide displayed selective cytotoxic activity against tumor cells, but is less active against non-tumor cell lines. Despite the powerful biological activity shown by TLMs, the biological mechanisms responsible for their action have never been elucidated. The objective of this study was to investigate, for the first time, the biological effects induced by thioalbamide in order to gain new insights on the TLMs’ mode of action. In particular, we explored the mechanisms of cellular death induced by this compound in breast cancer cell lines. Additionally, since we found that thioalbamide was able to impair mitochondrial bioenergetics, we investigated if it was able to target cancer stem-like cells (CSCs) considering that they are highly dependent on mitochondrial function for their clonal expansion and survival [9]. CSCs are a subset of cancer cells that are highly resistant to current therapeutic strategies, and are believed responsible for tumor recurrence and metastasis [10]. In this context, thioalbamide is revealed to be a promising natural agent that is able to affect tumorigenicity of breast CSCs.

## 2. Materials and Methods

### 2.1. Cell Cultures

All breast cell lines used in this work (MCF7, T47D, SKBR3, MDA-MB-231, MDA-MB-468, and MCF10A) were purchased from the American Culture Collection (ATCC, Manassas, VA, USA). Normal fibroblast BJ-hTERT cells were kindly provided by Professor Diego Sisci, University of Calabria, Italy. MCF7 and MDA-MB-231 cells were cultured in DMEM/F12 (Sigma Aldrich, St. Louis, MO, USA) supplemented with 10% Fetal Bovine Serum (FBS, Sigma Aldrich), 2 mM l-glutamine (Gibco, Life Technologies, Waltham, MA, USA), and 1% penicillin/streptomycin (Gibco, Life Technologies). MDA-MB-468 cells were cultured in DMEM (High Glucose) (Sigma Aldrich) supplemented with 10% FBS, 2 mM l-glutamine, and 1% penicillin/streptomycin. SKBR3 cells were cultured in RPMI supplemented with 10% FBS, 2 mM l-glutamine, and 1% penicillin/streptomycin. T47D cells were cultured in RPMI supplemented with 0.2 U/mL insulin (Gibco, Life Technologies) 10% FBS, 2 mM l-glutamine, and 1% penicillin/streptomycin. MCF10A cells were cultured in DMEM/F12 supplemented with 5% horse serum (HS, Sigma Aldrich), 10 mg/mL insulin (Sigma Aldrich), 0.5 mg/mL hydrocortisone (Sigma Aldrich), 20 ng/mL human epidermal growth factor (hEGF, Sigma Aldrich), 0.1 mg/mL cholera toxin (Sigma Aldrich), 2 mM l-glutamine, and 1% penicillin/streptomycin. BJ-hTERT were cultured in DMEM (Sigma Aldrich) supplemented with 1% penicillin/streptomycin, 2 mM l-glutamine, and 10% FBS. Treatments were performed in the above-mentioned media containing a lower amount of serum (2%). All cell lines were cultured at 37 °C in 5% CO_2_ in a humidified atmosphere. Thioalbamide was produced by the fermentation of *A. alba* DSM 44262 and then purified as described previously [1].

### 2.2. Cell Viability Assay

Cell viability was determined by using the 3-(4,5-dimethyl-2-thiazolyl)-2,5-diphenyl-2H-tetrazolium bromide (MTT) assay, as previously described [11]. Briefly, cells were seeded in 48-well plates with a density of 2 × 10^4^ cells/well and cultured in complete medium overnight. Cells were then treated with increasing concentrations of previously purified thioalbamide [1] or doxorubicin (Sigma Aldrich) for 72 h, and Dimethyl Sulfoxide (DMSO) was used as the vehicle control. At the end of the treatment, MTT solution was added to each well (to a final concentration of 0.5 mg/mL) and plates were incubated at 37 °C for 2 h until the formation of formazan crystals. DMSO-solubilized formazan in each well was quantified by absorbance at 570 nm using a microplate reader. Non-linear regression analysis (GraphPad Prism 7) was used to generate sigmoidal dose-response curves to calculate IC_50_ values for each cell line.

### 2.3. Cell Morphology Analysis

MCF7 cells were seeded into 6-well plates at a density of 1 × 10^5^ cells/well and cultured overnight in complete medium. Then, cells were treated with 50 nM thioalbamide for 72 h or DMSO (control cells), then cells were subjected to phase-contrast light microscopy analysis or fixed and stained with May Grünwald-Giemsa (Bio-Optica), as previously described [12]. Images at different magnifications were taken with an Olympus BX41 microscope with CSV1.14 software using CAMXC-30 for image acquisition.

### 2.4. Cell Cycle Analysis

Cell cycle analysis was performed as previously described [13] with minor changes. MCF7 cells, seeded in 6-well plates at a density of 1 × 10^5^ cells/well, were treated with DMSO or 50 nM thioalbamide for 72 h. After treatment, cells were collected by trypsinization, washed twice with chilled PBS, and spun at 1500 rpm for 5 min. Cells were fixed by re-suspending the pellets in 70% ethanol for 30 min at 4 °C. Cells were washed twice with PBS and stained in 3.8 mM sodium citrate, 50 µg/mL propidium iodide (PI), 100 µg/mL RNAse, and 0.1% Igepal in PBS for 1 h at 37 °C. Samples were subjected to cytofluorimetric analysis using BD FACSJazz™ Cell Sorter (Becton Dickinson, Franklin Lakes, NJ, USA).

### 2.5. Immunoblot Analysis

For immunoblot analysis, cells were grown to 70–80% confluence and treated with 50 nM thioalbamide or DMSO for 24–72 h. For preparation of total cell lysates, cells were harvested and lysed in 200 µL of lysis buffer, as previously described [14]. For cytosolic fraction isolation, cells were mechanically-lysed in isolation buffer for cells (IBC) (10 mM Tris/MOPS, 1 mM EDTA/Tris, 200 mM sucrose, pH 7.4) using a glass potter homogenizer and lysates were centrifuged at 12,000 rpm for 20 min. Supernatants and pellets were collected as cytosolic and mitochondrial fractions, respectively. The same amounts of proteins from total lysate or cytosolic fractions were resolved on SDS-polyacrylamide gel, transferred to a nitrocellulose membrane, and probed with appropriate primary antibodies (Santa Cruz, Biotechnology, Dallas, TX, USA). To confirm equal loading and transfer, membranes were stripped and incubated with the anti-GAPDH antibody (Santa Cruz, Biotechnology). The antigen-antibody complex was detected by incubation of the membranes with peroxidase-coupled goat anti-mouse or goat anti-rabbit antibodies and revealed using the ECL System (Bio-Rad Laboratories, Hercules, CA, USA) [15]. The blots were then exposed to film, and the bands of interest were quantified by using ImageJ software.

### 2.6. Terminal Deoxynucleotidyl Transferase-Mediated Deoxyuridine Triphosphate Nick End-Labeling (TUNEL) Assay

Fragmentation of DNA, a late event during apoptosis, was determined by enzymatic labelling of DNA strand breaks using terminal deoxynucleotidyl transferase-mediated deoxyuridine triphosphate nick end-labeling (TUNEL). TUNEL labeling was conducted using TUNEL assay kit (Promega, Madison, WI) on cells treated for 72 h with DMSO or 50 nM thioalbamide, according to the manufacturer’s instructions. Nuclear staining was performed by using 0.2 mg/mL 4′,6- diamidino-2-phenylindole (DAPI; Sigma Aldrich) and samples were analyzed with a fluorescent microscope (Olympus BX4 with CSV1.14 software using a CAMXC-30 for image acquisition).

### 2.7. AnnexinV-PI Assay

Phospatidilserine externalization on the outer leaflet of the cell membrane was detected by the AnnexinV-PI assay, according to the manufacturer’s protocols (Annexin V, Alexa Fluor^TM^ 488 conjugate kit, Thermo Fisher Scientific). Briefly, 1 × 10^5^ MCF7 cells were seeded in 6-well plates and treated with 50 nM thioalbamide for 24 h. After treatment, cells were collected, rinsed with PBS, and incubated with FITC-AnnexinV and 100 μg/mL propidium iodide in 1× AnnexinV buffer for 15 min at room temperature. After staining, samples were subjected to cytofluorimetric analysis by using the BD FACSJazz™ Cell Sorter (Becton Dickinson).

### 2.8. Mitochondrial Membrane Potential Analysis

To measure mitochondrial membrane potential, cells were stained with the CM-H2TMRos probe (MitoTracker Orange, ThermoFisher), whose accumulation in mitochondria is dependent upon membrane potential, according to the manufacturer’s protocols. Briefly, 1 × 10^5^ cells/well were seeded in 6-well plates and treated with 50 nM thioalbamide or DMSO for 72 h. After treatment, cells were harvested, rinsed, and incubated in 10 nM MitoTracker Orange solution in PBS for 30 min at 37 °C. After staining, cells were fixed with 3.7% formaldehyde in PBS at 37 °C for 15 min, rinsed, and resuspended in PBS. Samples were then subjected to cytofluorimetric analysis by using the BD FACSJazz™ Cell Sorter (Becton Dickinson). Cells incubated with 2 μM FCCP for 30 min were used as a positive control for mitochondrial membrane potential reduction.

### 2.9. Reactive Oxygen Species (ROS) Assessment

1 × 10^5^ MCF7 cells/well were seeded in 6-well plates and treated with 50 nM thioalbamide or DMSO for 24/72 h. Cells treated with 1 mM H_2_O_2_ for 6 h were used as the positive control. After treatment, cells were washed with PBS, collected, resuspended in 5 μM CM-H_2_DCFDA (ThermoFisher Scientific) in PBS, and incubated for 45 min at 37 °C. Stained cells were collected by centrifugation, and resuspended in fresh pre-chilled medium. Finally, the fluorescence of samples was quantified with a fluorimeter (Synergy H1 microplate reader, BioTek, Winooski, VT, USA), and fluorescence intensity was normalized by viable cell number (TC20 automated cell counter, Bio-Rad).

### 2.10. ROS-Scavenging Assay

To evaluate if thioalbamide cytotoxicity was mediated by oxidative stress, cells were treated with 50 nM thioalbamide in the presence or absence of 1 mM vitamin E or 600 μM N-acetyl cysteine (NAC) for 72 h. After treatment, cell viability was assessed by the MTT assay, as described above in the cell viability section.

### 2.11. Superoxide Dismutase Activity Assay

Superoxide dismutase activity was assessed on total cell lysates (50 μg of proteins) by using a colorimetric SOD assay kit (Sigma Aldrich) as per the manufacturer’s instructions. This is an indirect assay method based on xanthine oxidase mediated production of superoxide anion that is able to reduce 2-(4-iodophenyl)-3-(4-nitrophenyl)-5-(2,4-disulfophenyl)-2H-tetrazolium monosodium salt (WST-1) in a water-soluble formazan dye. SOD activity, which neutralizes superoxide anions, inhibits WST-1 reduction and dye formation that can quantified by monitoring absorbance at 450 nm using a microplate reader (Synergy H1 microplate reader, BioTek). SOD1 enzyme (Sigma-Aldrich) was used as a standard.

### 2.12. Seahorse XFe96 Metabolic Profile Analysis

Real-time oxygen consumption rates (OCR) and extracellular acidification rates (ECAR) rates for cells treated with thioalbamide were determined using the Seahorse Extracellular Flux (XFe96) analyzer (Seahorse Bioscience, USA) to assess mitochondrial and glycolytic function, respectively. The analysis was performed as previously described [16]. Briefly, 1 × 10^4^ cells per well were seeded into XFe96 well cell culture plates, and incubated overnight to allow for cell attachment. Then, cells were treated with thioalbamide (50 nM and 100 nM) for 48 h. Vehicle alone (DMSO) control cells were processed in parallel. After 48 h of incubation, cells were washed in pre-warmed XF assay media (or for OCR measurement, XF assay media supplemented with 10 mM glucose, 1 mM Pyruvate, 2 mM l-glutamine, and adjusted at pH 7.4). Cells were then maintained in 175 µL/well of XF assay media at 37 °C in a non-CO_2_ incubator for 1 h. During the incubation time, we loaded 25 µL of 80 mM glucose, 9 µM oligomycin, and 1 M 2-deoxyglucose (for ECAR measurement) or 10 µM oligomycin, 9 µM FCCP, 10 µM rotenone, 10 µM antimycin A (for OCR measurement) in the XF assay media into the injection ports in the XFe96 sensor cartridge. Measurements were normalized by protein content (SRB assay). Datasets were analyzed using XFe96 software and GraphPad Prism software through one-way ANOVA and Student’s *t*-test calculations. All experiments were performed in quintuplicate, three times independently.

### 2.13. Mammosphere Formation Assay

A single cell suspension was prepared using enzymatic (1x Trypsin-EDTA, Sigma Aldrich), and manual disaggregation (25 gauge needle). Cells were seeded at a density of 5000 cells/well in a mammosphere medium (DMEM-F12 + B27 supplement + 20 ng/mL EGF + penicillin/streptomycin) under non-adherent conditions in 6-well plates pre-coated with (2-hydroxyethylmethacrylate) (poly-HEMA, Sigma Aldrich). Then, cells were treated with thioalbamide at concentrations ranging from 25 nM to 100 nM. Vehicle alone (DMSO) treated cells were processed in parallel as a control. Cells were grown for five days and maintained in a humidified incubator at 37 °C. After five days of culture, 3D-spheres >50 μm were counted using an eye piece (“graticule”), and the percentage of cells plated that formed spheres was calculated and is referred to as the percent mammosphere formation efficiency (MFE).

### 2.14. ALDEFLUOR Assay

The levels of ALDH enzymatic activity in cell sub-populations were assessed using the fluorescent reagent ALDEFLUOR kit (StemCell Technologies, Durham, NC, USA) by FACS (SONY SH800). Briefly, 1 × 10^5^ cells were incubated in 1 mL ALDEFLUOR assay buffer containing ALDH substrate (5 μL/mL) for 40 min at 37 °C. Cells isolated from mammospheres were trypsinized and syringed twice with a 25G needle before incubation and analysis. For each experiment, a sample of cells was stained under identical conditions in the presence of 30 μM diethylaminobenzaldehyde (DEAB), a specific ALDH inhibitor, as a negative control. The ALDH-positive population was established according to the manufacturer’s instructions and evaluated using 50,000 cells (for adherent cells) and 30,000 (for mammospheres). All of the ALDH experiments were performed three times independently.

### 2.15. CD44 Expression

CD44 expression was evaluated in cells in grown in adherent conditions as well as from cells isolated from mammospheres. For adherent conditions, 1 × 10^5^ cells were treated with 50 nM thioalbamide for 72 h in 6-well plates, grown as a monolayer. Cells were spun down and incubated with the CD44 antibody (APC mouse Anti-Human CD44, BD Pharmingen cat. 559942) for 15 min on ice in DPBS Ca^2+^/Mg^2+^. For mammosphere conditions, cells derived from mammospheres were spun down and syringed twice in the presence of trypsin 0.5% using a 25G needle. 1 × 10^5^ MCF7 cells were incubated with the CD44 (APC mouse Anti-Human CD44, BD Pharmingen cat. 559942) antibody for 15 min on ice in DPBS Ca^2+^/Mg^2+^. Samples were analyzed by FACS (SONY SH800) and data were analyzed using FlowJo software.

### 2.16. Statistical Analysis

Data are presented as the mean values ± standard deviation, taken over ≥3 independent experiments, with ≥3 replicates per experiment, unless otherwise stated. Statistical significance was measured by using the analysis of variance (ANOVA) test. A *P* value ≤ 0.05 was considered statistically significant. Non-linear regression analysis (GraphPad Prism 7) was used to generate sigmoidal dose-response curves to calculate the IC_50_ values.

## 3. Results

### 3.1. Thioalbamide Affects Cell Viability of Several Breast Cancer Cellular Lines

The antiproliferative effects of thioalbamide were evaluated over a wide range of breast cancer cell lines characterized by differences in the status of the three main receptors conventionally used for breast cancer subtyping: estrogen receptor (ER), progesterone receptor (PR), and human epithelial receptor 2 (HER2) [17]. In particular, we evaluated the effects of thioalbamide on the viability of luminal cell lines MCF7 and T47D in the HER2 positive cell line SKBR3 and in triple-negative cell lines MDA-MB-231 and MDA-MB-468. Thioalbamide showed a significant inhibitory effect on the viability of all lines tested. The IC_50_ values, calculated for each experimental model after 72 h of treatment, were all in a range between 54 and 75 nM (Table 1). The effects on cell viability were significantly higher than the clinically used doxorubicin (IC_50_ values ranging from 154 nM to 1.17 µM), and were notably independent of the receptor profile of mammary tumor cells.

Importantly, thioalbamide did not exert any effects on the viability of the non-malignant breast epithelial cell line MCF10A and the normal fibroblast cell line BJ-H, at concentrations toxic for cancer cells. As reported in Table 1, the IC_50_ value for MCF10A cells was up to 6-fold higher than those for the tumor cell lines, highlighting thioalbamide’s specificity for the malignant phenotype. Since thioalbamide has shown to possess anti-viability activity at very similar concentrations in all tumor cell lines tested, independently of their tumor subtype, we decided to focus the mechanistic studies on a single experimental model, the luminal breast carcinoma cell line MCF7.

### 3.2. Thioalbamide Induces Cellular Morphology Changes

**Figure 2:**
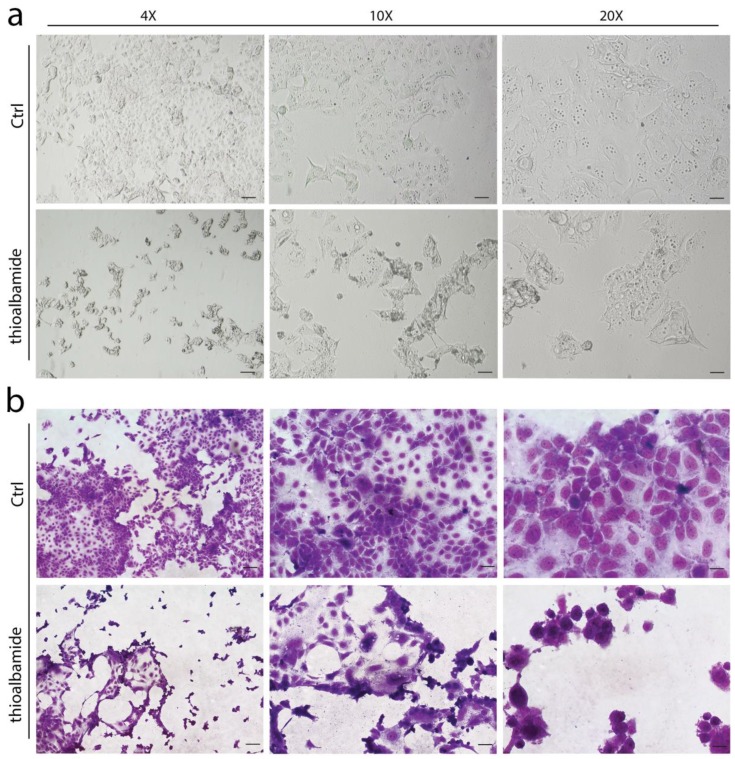
Thioalbamide affects cell morphology. MCF7 cells were treated with DMSO (Ctrl) or 50 nM thioalbamide, for 72 h, and cell morphology was evaluated by phase-contrast light microscopy (**a**) and May-Grunwald Giemsa staining (**b**). Scale bars: 4× (125 µm), 10× (50 µm), 20× (25 µm). Note that cell morphology after treatment was seriously affected.

First, the ability of thioalbamide to induce morphological changes in the treated cells was evaluated. As highlighted by phase-contrast light microscopy as well as by May-Grunwald and Giemsa staining, after 72 h of treatment, the MCF7 cell morphology was drastically altered, and the treated cells appeared more heterogeneous in terms of shape and size. As shown in Figure 2, the untreated control cells grew well and exhibited epithelial-like features, forming a monolayer on the plate. Conversely, the growth of MCF7 treated with thioalbamide was reduced, and cell fusion, shrinkage, nuclear condensation, and apoptotic bodies appeared.

### 3.3. Thioalbamide Induces Arrest of Cell Cycle in G1 Phase

**Figure 3:**
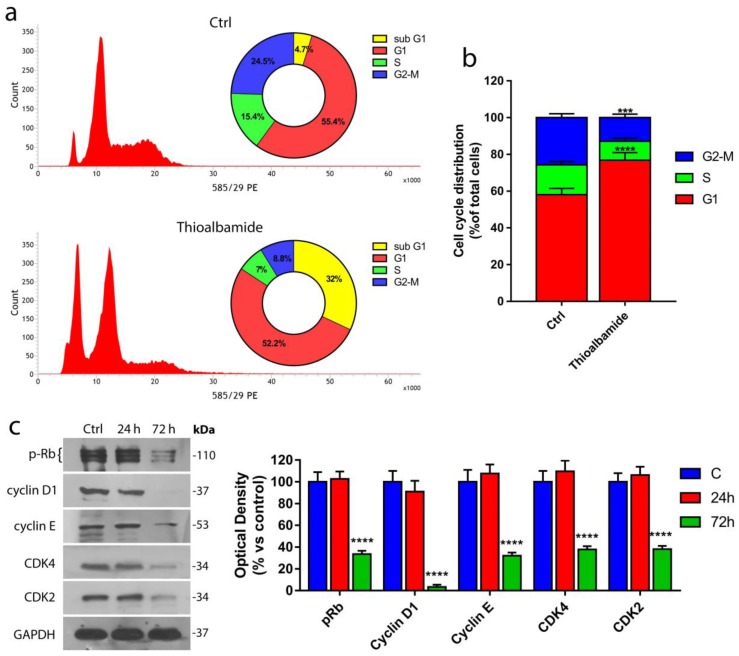
Thioalbamide inhibits cell cycle progression. (**a**) Cytofluorimetric analysis of MCF7 cells treated for 72 h with DMSO (control) or 50 nM thioalbamide and stained with PI; graphs represent the percentage of total events in the samples (highlighting the sub-G1 peak). (**b**) Cell cycle distribution of MCF7 cells (excluding sub-G1 events). (**c**) Immunoblot analysis of the main regulators of G1/S phase transition and quantification of their expression levels by densitometry. Note that thioalbamide treatment induces cell cycle arrest at the G1/S checkpoint. Values represent mean ± SD of three independent experiments. **** *P* value < 0.0001.

The effects of thioalbamide on viability were further investigated. In particular, the ability of thioalbamide to alter the different phases of the cell cycle was evaluated using cytofluorimetric analysis. As shown in Figure 3a,b, thioalbamide treatment induced a substantial decrease of cells in the S and G2-M phases, together with the appearance of a significant hypodiploid population (sub-G1). The peak of hypodiploid cells is generally associated with cells undergoing apoptosis, where genomic DNA appears drastically fragmented [18,19]. These results suggest that thioalbamide treatment can block the transition from G1 to the subsequent phases of the cell cycle, and cells accumulate in the G1 phase before undergoing cell death.

These results were confirmed by the immunoblotting analysis of several proteins involved in the G1-S transition (Figure 3b,c) such as cyclins E and D1 as well as their respective cyclin-dependent kinase (CDK2 and CDK4), with which they are able to form active complexes [20,21]. Our findings showed a drastic reduction in both CDKs and their respective cyclins in MCF7 cells after 72 h of thioalbamide treatment. Cell cycle arrest in the G1 phase was also confirmed by the dephosphorylation and activation of Retinoblastoma protein (Rb), an oncosuppressor able to bind to the E2F transcription factor family [22,23], inhibiting their function and DNA replication.

### 3.4. Thioalbamide Induces Cell Death by Activation of both Extrinsic and Intrinsic Apoptotic Pathways

**Figure 4:**
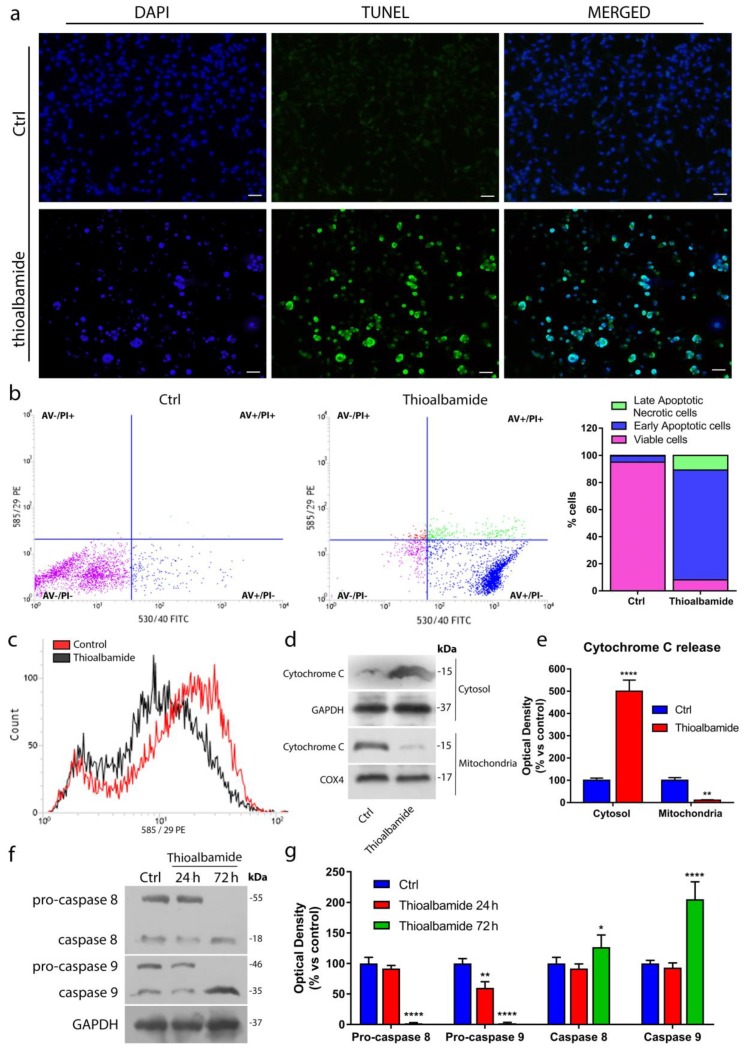
Thioalbamide induces cell death by activation of both extrinsic and intrinsic apoptotic pathways. (**a**) TdT-mediated dUTP nick-end-labeling (TUNEL) assay in MCF7 cells treated for 72 h with vehicle (Ctrl) or thioalbamide. DAPI was used for DNA staining (scale bar: 50 μm). (**b**) Annexin V-PI assay was performed to detect apoptosis in DMSO-treated cells (Ctrl) and cells treated with thioalbamide for 24 h; histograms represent the percentage of cell populations after treatment. (**c**) Loss of mitochondrial membrane potential after 72 h of thioalbamide treatment was assessed using a MitoTracker Orange CM-H2TMRos probe. (**d**) Release of cytochrome C into the cytosolic and mitochondrial fractions of cells treated for 72 h with thioalbamide was assessed by immunoblot analysis. GAPDH and COX4 were used as equal loading controls of the cytosolic and mitochondrial fractions, respectively. (**e**) Quantification of cytochrome C levels in the cytosolic and mitochondrial fractions by densitometry. (**f**) Immunoblot analysis of pro-caspases 8 and 9 after 24 and 72 h of treatment with thioalbamide. GAPDH was used as the equal loading control. (**g**) Quantification of pro-caspase expression levels by densitometry. All thioalbamide treatments were performed at 50 nM. Note that thioalbamide treatment triggers early and late events of apoptosis, involving both intrinsic and extrinsic pathways. Values represent the mean ± SD of three independent experiments. * *P* value < 0.05; ** *P* value < 0.01; **** *P* value < 0.0001.

Thioviridamide, the first identified molecule of the TLMs family, is known to induce cell death through apoptotic processes [4]. This knowledge, together with the evidence that thioalbamide treatment is able to enrich the hypodiploid cell population, has led us to investigate whether the trigger of apoptosis is a common feature of thioviridamide-like molecules. For this purpose, MCF7 cells were treated with thioalbamide and subjected to different assays, each one specific for a different event of the apoptotic pathway. The TUNEL assay was performed to highlight the ability of thioalbamide to induce DNA fragmentation, a late event in apoptosis. As shown in Figure 4a and Figure S6, after 72 h of treatment, the percentage of TUNEL-positive cells was elevated, which is consistent with the increased population of hypodiploid cells observed in the cell cycle.

Next, Annexin V assay was performed to monitor the externalization of phosphatidylserine on the outer leaflet of the cell membrane. This event characterizes the early stage of apoptosis and, in physiological conditions, facilitates apoptotic cells recognition from macrophages and destruction of apoptotic bodies [24,25]. Interestingly, 24 h of thioalbamide treatment were sufficient for 80% of the treated cells to enter an early stage of apoptosis (Figure 4b). In order to definitively confirm the induction of the apoptotic process, the ability of thioalbamide to determine a loss of mitochondrial membrane potential and the release of cytochrome C, normally confined within mitochondria, into the cytosol were evaluated. The loss of mitochondrial membrane potential represents the “point of no-return” in the apoptotic process. Indeed, this event is responsible for a change in mitochondrial membrane permeability and leads to the translocation into the cytoplasm of cytochrome C that, by forming apoptosome complex, triggers the apoptotic cascade of caspases [26]. After treatment, MCF7 cells showed a significant reduction in mitochondrial membrane potential with respect to untreated cells (Figure 4c, Figure S9). Moreover, immunoblotting analysis of cytochrome C levels in the cytosolic fraction confirmed that thioalbamide induced cytochrome C release, with a concomitant reduction of cytochrome C found into the mitochondrial fraction (Figure 4d,e). Finally, it was decided to evaluate the cleavage and activation of procaspases -8 and -9, initiator caspases involved in extrinsic and intrinsic apoptosis pathways respectively [27]. Immunoblot analysis revealed that thioalbamide treatment induced the activation of both pathways in MCF7 cells (Figure 4f,g).

In order to understand if the apoptotic mechanisms induced by thioalbamide observed in MCF7 cells, are also underlying the cytotoxic effects observed in the other breast cancer cells, TUNEL assay was performed in MDA-MB-231, MDA-MB-468, T47D and SKBR3 cells treated with thioalbamide. Our results indicate that thioalbamide induces cell death via apoptosis in all tested breast cancer cell lines (Figures S2–S6).

### 3.5. Oxidative Stress Underlies the Cytotoxic Effects of Thioalbamide

**Figure 5:**
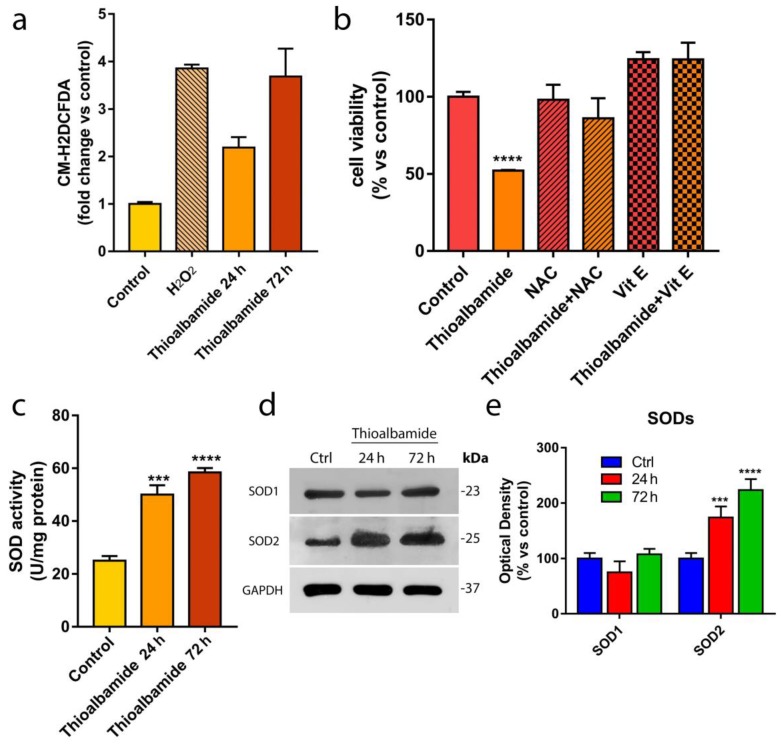
Oxidative stress underlies the cytotoxic effects of thioalbamide. (**a**) ROS intracellular levels were measured after thioalbamide treatment of MCF7 cells for 24 and 72 h. Results obtained from cells treated for 6 h with 1 mM H_2_O_2_, which was used as the positive control, were related to their own control. (**b**) MCF7 cell viability was assessed after treatment for 72 h with thioalbamide alone or in combination with vitamin E (Vit E) or N-acetyl cysteine (NAC). (**c**) Total SOD activity assay was performed on MCF7 cells treated with thioalbamide for 24 and 72 h. (**d**) Immunoblot analysis of SOD1 and SOD2 expression levels in MCF7 cells treated with thioalbamide for 24 and 72 h. GAPDH was used as equal loading control. (**e**) Quantification of SOD expression levels by densitometry. Thioalbamide treatments were performed at 50 nM. Note that ROS accumulation and oxidative stress underlie the proapoptotic activity of thioalbamide. Values represent the mean ± SD of three independent experiments. *** *P* value < 0.001; **** *P* value < 0.0001.

In order to better understand the mechanisms underlying thioalbamide-induced apoptosis, the levels of reactive oxygen species (ROS) produced in untreated and treated MCF7 cells were assessed by cytofluorimetric analysis. ROS are well known biochemical mediators of apoptosis as they are strongly reactive toward several biological macromolecules, particularly toward proteins involved in the electro-chemical equilibrium of the mitochondrial membrane [28]. Interestingly, thioalbamide induced a significant 2-fold increase in ROS levels after 24 h of treatment, and ROS reached a 4-fold increase after 72 h of treatment, relative to the control cells (Figure 5a).

In order to confirm that oxidative stress is responsible for thioalbamide-induced cytotoxicity, a viability assay was performed on MCF7 treated with thioalbamide alone and in the presence of a ROS scavenger such as vitamin E and N-acetyl cysteine. After 72 h of treatment, no cytotoxicity was detected in the thioalbamide-scavenger co-treated cells (Figure 5b), confirming that oxidative stress is the event responsible for the induction of apoptosis and cytotoxicity. The same result was obtained in all of the other cancer cell lines tested, demonstrating that oxidative stress induced by thioalbamide is responsible for the cytotoxic effects observed in all of the different breast cancer cell lines used in this study (Figure S7).

In order to understand whether thioalbamide-induced ROS accumulation is due to an effective increase in ROS production or to a deficiency of the ROS neutralization system, the expression levels and activity of superoxide dismutases (SODs) were evaluated in MCF7 cells treated with thioalbamide. The SOD family of enzymes are specifically responsible for the neutralization of superoxide anion (**^.^**O_2_^−^), one of the main sub-products of cellular metabolism that originates from an incomplete reduction of molecular oxygen in the mitochondrial respiratory chain [29]. Interestingly, SOD activity was increased by >2 folds in cells treated with thioalbamide for 72 h as a physiological response to the increased ROS production (Figure 5c). However, the increase in SOD activity was not sufficient to balance the time-dependent ROS accumulation induced by thioalbamide treatment.

There are several isoforms of SOD that are specific for different cellular compartments [30]. Thus, immunoblotting analysis of the different SOD isoforms was performed in order to understand whether the increase in SOD activity observed was specific to a cellular compartment. Figure 5d–e showed that thioalbamide treatment increased the expression of SOD2, the mitochondrial isoform, while the levels of SOD1, the cytoplasmic isoform, were found to be unaltered. These results indicate an increase in ROS production at the mitochondrial level [31], suggesting an important involvement of mitochondria in thioalbamide’s mechanism of action.

### 3.6. Thioalbamide Treatment Affects Glycolysis and Mitochondrial Respiration

It is known that the production of ROS is closely related to cellular energy metabolism [32]. Therefore, the ability of thioalbamide treatment to alter the breast cancer cell metabolic profile was evaluated by assessing mitochondrial respiration and glycolytic function by using a Seahorse XFe96 analyzer.

**Figure 6:**
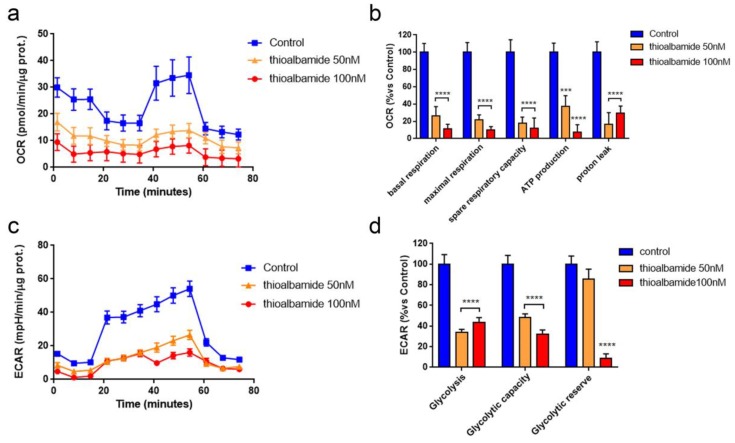
Thioalbamide inhibits metabolic functions in MCF7 cells. A Seahorse XFe96 analyzer was employed to analyze mitochondrial respiration and glycolytic function in MCF7 cells treated with thioalbamide for 48 h by monitoring oxygen consumption rate (OCR) and the extracellular acidification rate (ECAR), respectively. (**a**) OCR representative tracings of MCF7 cells treated with DMSO (control) or 50–100 nM thioalbamide. (**b**) Histograms of mitochondrial respiration parameters. (**c**) ECAR representative tracings of MCF7 cells treated with DMSO (control) and 50–100 nM thioalbamide. (**d**) Histograms of glycolytic function parameters. Significant reductions of mitochondrial and glycolytic function were observed experimentally. Values represent the mean ± SEM of five biological replicates of three independent experiments. *** *P* value < 0.001; **** *P* value < 0.0001.

Mitochondrial function was assessed by monitoring oxygen consumption rate (OCR), and sequentially, injections of oligomycin, FCCP, and an antimycin/rotenone mix allowed us to evaluate mitochondrial function parameters such as basal respiration, maximal respiration, ATP production, proton leak, and spare respiratory capacity. Interestingly, MCF7 cells treated with thioalbamide for 48 h displayed a dramatic reduction of all mitochondrial respiration parameters (Figure 6a,b), which is consistent with the above-mentioned reduction in mitochondrial membrane potential (Figure 4c).

Glycolytic function was assessed by monitoring extracellular acidification rate (ECAR), and sequentially, injections of glucose, oligomycin, and 2-deoxy-D-glucose (2-DG) allowed us to evaluate the glycolytic function parameters. Notably, MCF7 cells treated with thioalbamide for 48 h showed significantly reduced glycolysis and glycolytic capacity, while the glycolytic reserve was affected only at higher concentrations (Figure 6c,d). Similar results with the metabolic profile and mitochondrial membrane potential were obtained in the other breast cancer lines tested (Figures S8 and S9), emphasizing once again that the effects induced by thioalbamide are similar in all breast cancer cell lines, independent of their subtype and biological heterogeneity.

Overall, our results highlight the ability of thioalbamide to inhibit energy metabolism of breast cancer cells, affecting the two main pathways of cell metabolism: glycolysis and oxidative phosphorylation. These effects result in a significant change in the metabolic phenotype of cancer cells from a highly energy status to a quiescent one.

### 3.7. Thioalbamide Affects Breast Cancer Stem Cells Propagation

**Figure 7:**
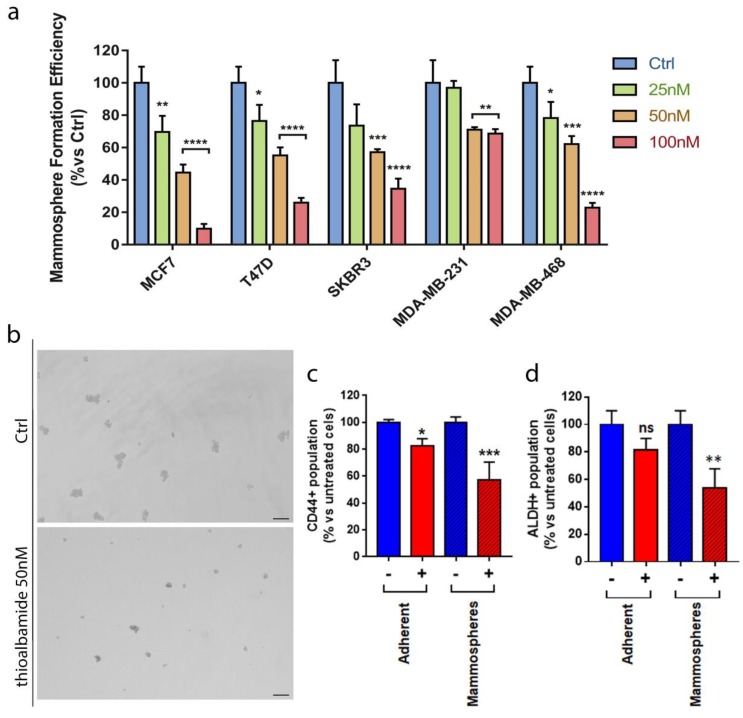
Thioalbamide inhibits CSCs propagation. (**a**) Mammosphere formation efficiency assay was performed on breast cancer cells (MCF7, T47D, SKBR3, MDA-MB-231, and MDA-MB-468) after treatment for five days with 25 nM, 50 nM, and 100 nM thioalbamide. All breast cancer cells tested showed a significant dose dependent reduction in mammosphere formation after treatment with thioalbamide. Mammospheres were grown in no-attachment conditions and counted using an Olympus BX41 (4× magnification) microscope. 3D-spheres >50 μm were counted using an eye piece (“graticule”), and the percentage of cells plated that formed spheres was calculated and referred to as percent mammosphere formation efficiency (MFE). (**b**) Representative images of MCF7 cell mammospheres. Scale bar: 125 µm. (**c**) CD44 expression and (**d**) ALDH activity were chosen as CSC markers. Anti-human CD44-APC antibodies and (**d**) an ALDEFLUOR assay kit were used to stain MCF7 cells. Samples were analyzed using a SONY SH800 cell sorter. A significant reduction of CD44-positive cells and ALDH-positive cells was detected after treatment with 50 nM of thioalbamide in MCF7 cells grown in no-attachment (mammospheres) conditions. Values represent the mean ± SD of three independent experiments. ns = not significant; * *P* value < 0.05; ** *P* value <0.01; *** *P* value < 0.001; **** *P* value < 0.0001.

In recent years, much focus has been given to the identification of molecules that are able to inhibit cancer stem cells (CSCs), the sub-population of tumor cells responsible for therapy resistance and tumor recurrence. Since mitochondrial function is required for anchorage-independent survival and propagation of cancer stem-like cells [33], and considering that thioalbamide strongly reduces cancer cell mitochondrial function, we investigated if thioalbamide may affect cancer stem cells. To this end, we monitored mammosphere formation efficiency (MFE) as well as two validated CSC markers, the activity of aldehyde dehydrogenase (ALDH) and the expression of CD44 antigen. The results obtained indicate that thioalbamide is able to reduce, in a dose-dependent manner, mammosphere formation efficiency in all the cell lines tested (Figure 7a,b) as well as the ALDH+ population in MCF7-derived spheroids (Figure 7c) and CD 44 expression in adherent cells and mammospheres (Figure 7d and Figure S10). These results demonstrate a significant inhibition of breast cancer stem cells, confirming thioalbamide inhibitory effects on cell metabolism and opening new scenarios regarding the use of this natural product in the oncologic field.

## 4. Discussion

Since the discovery of penicillin by Alexander Fleming in 1928, microbial-derived natural products have been an essential resource for the development of new pharmacological agents [34]. Within the wide variability of microorganisms populating terrestrial and marine ecosystems, bacteria belonging to the phylum *Actinobacteria* are the main source of natural bioactive molecules [35,36]. Indeed, the secondary metabolism of these microorganisms is complex and extremely variable, and is responsible for the production of very different molecules from a chemical, biosynthetic. and biological activity point of view [2,37,38,39].

Thioviridamide-like molecules are a family of thioamided peptides with a high antitumor potential that appear to be highly conserved, despite the profound variability between the amino acid sequence of the different family members [8]. Although in recent years various advances have been made on the identification of new TLMs [7] and in understanding the biosynthetic pathways underlying their production in *Actinobacteria* [40,41], no study has so far been carried out to elucidate the molecular and cellular mechanisms underlying their enormous pharmacological potential. Herein, a biochemical-metabolic approach was used to investigate in different breast cancer cell lines the mechanism of action of thioalbamide, a TLM, which in previous studies has demonstrated a strong cytotoxic activity highly specific toward malignant phenotypes [1].

In this study, we used a large panel of breast cancer cells to reflect the biological diversity of the different breast cancer subtypes, characterized by a different response to traditional chemotherapy, endocrine therapy, and immunotherapy [42,43]. Despite the effectiveness of these therapeutic approaches and their widespread use, the onset of resistance and recurrence remains an unmet clinical need, driving the continuous research for alternative pharmaceutical approaches. Our results, obtained on MCF7, T47D, SKBR3, MDA-MB-231, and MDA-MB-468 cells, underline the independence of the thioalbamide mode of action from the receptor status of the tumor cells, highlighting the profound versatility of this microbial natural peptide.

Using microscopy, cytofluorimetric, and immuno-detection analysis, we demonstrated that thioalbamide induced cell cycle arrest in the G1 phase and cell death by intrinsic and extrinsic apoptotic mechanisms. Pro-apoptotic effects had been previously evidenced for thioviridamide [4], the first TLM identified, and our results confirm that these mechanisms are common among different members of the family. This evidence suggests that, despite the variability of the amino acid residues that make up these metabolites, the conserved chemical characteristics of these natural products lie at the basis of their enormous antitumor potential. The arrest of the cell cycle in the G1 phase, moreover, suggests that thioalbamide can affect the normal physiological processes of the cell [44] that does not meet the replicative processes and subsequently dies.

In addition, we discovered that an increase in ROS production is the trigger for thioalbamide-induced apoptotic cell death. Excessive increase in intracellular ROS levels induced by thioalbamide treatment greatly affects mitochondrial function. Indeed, breast cancer cells respond to thioalbamide-induced oxidative stress with a selective increase in the activity and expression levels of the mitochondrial isoform of superoxide dismutase (SOD2), an enzyme normally responsible for neutralizing superoxide anions, the main sub-products of cellular respiration.

Since ROS are generated by cellular metabolism, their accumulation and consequent oxidative stress are often associated with alterations to metabolic pathways [45]. Metabolic reprogramming is one of the characteristics of cancer, since tumors require catabolites to produce ATP, maintain a redox balance, and generate biomass. Depending on the availability of nutrients, some cells within the tumor are predominantly glycolytic, while others have a phenotype dependent on oxidative phosphorylation. Since the metabolic reprogramming of tumor cells accelerates energy metabolism to support the high proliferation rate of malignant neoplasms [46], metabolic processes represent an important pharmacological target for cancer therapy. Therefore, in this work, the energy profile of cells treated with thioalbamide was also evaluated, and the results highlight the ability of this natural product to inhibit the two main cellular energy pathways: glycolysis and oxidative phosphorylation. It is known that under physiological conditions, a reduction in mitochondrial respiration leads to a compensatory increase in glycolytic metabolism, which is fundamental for maintaining cellular energy requirements [47,48]. However, in our experimental conditions, the thioalbamide effects are consistent with what has been reported in other studies, showing that OXPHOS inhibitors [49] and oxidative stress inducers [50] generate only a temporary spike of glycolytic metabolism, while a prolonged exposure to such chemicals causes a drastic reduction of the extracellular acidification rate.

Moreover, we show here that thioalbamide can inhibit the growth and propagation of cancer stem-like cells (CSCs), the subpopulation of tumor cells mainly responsible for cancer resistance, recurrence, and metastasis. CSCs are characterized by a metabolic profile that differentiates them from bulk tumor cells and healthy stem cells. Indeed, CSCs have a high metabolic flexibility that allows them to switch between the glycolytic and phosphorylative pathways, according to the energy needs of the malignant cell. Indeed, our results indicate that thioalbamide may eradicate CSCs by targeting their energy metabolism and reducing the metabolic flexibility responsible for their tumorigenicity [51,52,53].

As such, the effects of thioalbamide on CSCs opens new avenues in the application of this microbial natural product in cancer therapy. Further studies will be needed to better delineate the exact molecular mechanism underlying the biological activity of thioalbamide and TLMs. Furthermore, in vivo pharmacokinetic and pharmacodynamic studies are indispensable to further explore the promising potential of this new family of microbial natural products in more complex experimental models to validate the potential use of thioalbamide in clinical settings.

## Figures and Tables

**Table 1:** Cytotoxic activity of thioalbamide in comparison to doxorubicin ^a^.

Cell Line		Doxorubicin	Thioalbamide
MCF7 ^b^	IC_50_ (μM)	0.878	0.059
	95% confidence interval	0.723 to 1.071	0.049 to 0.072
MDA-MB-231 ^b^	IC_50_ (μM)	1.174	0.072
	95% confidence interval	0.938 to 1.477	0.058 to 0.088
MDA-MB-468	IC_50_ (μM)	0.362	0.054
	95% confidence interval	0.259 to 0.504	0.043 to 0.069
T47D	IC_50_ (μM)	0.536	0.075
	95% confidence interval	0.380 to 0.756	0.061 to 0.092
SKBR3	IC_50_ (μM)	0.154	0.074
	95% confidence interval	0.108 to 0.221	0.056 to 0.097
MCF 10A ^b^	IC_50_ (μM)	0.343	0.302
	95% confidence interval	0.253 to 0.464	0.206 to 0.444
BJ-hTERT	IC_50_ (μM)	0.360.5	0.321
	95% confidence interval	0.269 to 0.484	0.215 to 0.474

^a^ Data are presented as IC_50_ values (μM) and 95% confidence intervals obtained by nonlinear regression analysis of three independent experiments. ^b^ Results reported in [1].

## Supplementary Materials

The following are available online at https://www.mdpi.com/2073-4409/8/11/1408/s1: Figure S1: Thioalbamide affects breast cancer cellular growth; Figure S2: Thioalbamide-induced DNA fragmentation in MDA-MB-468 cells; Figure S3: Thioalbamide-induced DNA fragmentation in SKBR3 cells; Figure S4: Thioalbamide-induced DNA fragmentation in T47D cells; Figure S5: Thioalbamide-induced DNA fragmentation in MDA-MB-231 cells; Figure S6: Thioalbamide induces apoptosis in breast cancer cell lines; Figure S7: Oxidative stress underlies thioalbamide cytotoxicity in breast cancer cell lines; Figure S8: Metabolic profile of breast cancer cells treated with thioalbamide; Figure S9: Thioalbamide induces loss of mitochondrial membrane potential in breast cancer cells; Figure S10: Thioalbamide reduces CD44 expression in breast cancer cells.

## References

[1] Frattaruolo L., Lacret R., Cappello A.R., Truman A.W. (2017). A Genomics-Based Approach Identifies a Thioviridamide-Like Compound with Selective Anticancer Activity. Acs Chem. Biol..

[2] Arnison P.G., Bibb M.J., Bierbaum G., Bowers A.A., Bugni T.S., Bulaj G., Camarero J.A., Campopiano D.J., Challis G.L., Clardy J. (2013). Ribosomally synthesized and post-translationally modified peptide natural products: Overview and recommendations for a universal nomenclature. Nat. Prod. Rep..

[3] Kjaerulff L., Sikandar A., Zaburannyi N., Adam S., Herrmann J., Koehnke J., Muller R. (2017). Thioholgamides: Thioamide-Containing Cytotoxic RiPP Natural Products. Acs Chem. Biol..

[4] Hayakawa Y., Sasaki K., Adachi H., Furihata K., Nagai K., Shin-ya K. (2006). Thioviridamide, a novel apoptosis inducer in transformed cells from Streptomyces olivoviridis. J. Antibiot..

[5] Dunbar K.L., Scharf D.H., Litomska A., Hertweck C. (2017). Enzymatic Carbon–Sulfur Bond Formation in Natural Product Biosynthesis. Chem. Rev..

[6] Santos-Aberturas J., Chandra G., Frattaruolo L., Lacret R., Pham T.H., Vior N.M., Eyles T.H., Truman A.W. (2019). Uncovering the unexplored diversity of thioamidated ribosomal peptides in Actinobacteria using the RiPPER genome mining tool. Nucleic Acids Res..

[7] Tang J., Lu J., Luo Q., Wang H. (2018). Discovery and biosynthesis of thioviridamide-like compounds. Chin. Chem. Lett..

[8] Kudo K., Koiwai H., Kagaya N., Nishiyama M., Kuzuyama T., Shin-Ya K., Ikeda H. (2019). Comprehensive Derivatization of Thioviridamides by Heterologous Expression. Acs Chem. Biol..

[9] Fiorillo M., Lamb R., Tanowitz H.B., Mutti L., Krstic-Demonacos M., Cappello A.R., Martinez-Outschoorn U.E., Sotgia F., Lisanti M.P. (2016). Repurposing atovaquone: Targeting mitochondrial complex III and OXPHOS to eradicate cancer stem cells. Oncotarget.

[10] Fiorillo M., Peiris-Pages M., Sanchez-Alvarez R., Bartella L., Di Donna L., Dolce V., Sindona G., Sotgia F., Cappello A.R., Lisanti M.P. (2018). Bergamot natural products eradicate cancer stem cells (CSCs) by targeting mevalonate, Rho-GDI-signalling and mitochondrial metabolism. Biochim. Biophys. Acta. Bioenerg..

[11] Perri F., Frattaruolo L., Haworth I., Brindisi M., El-magboub A., Ferrario A., Gomer C., Aiello F., Adams J.D. (2019). Naturally occurring sesquiterpene lactones and their semi-synthetic derivatives modulate PGE2 levels by decreasing COX2 activity and expression. Heliyon.

[12] Bonesi M., Brindisi M., Armentano B., Curcio R., Sicari V., Loizzo M.R., Cappello M.S., Bedini G., Peruzzi L., Tundis R. (2018). Exploring the anti-proliferative, pro-apoptotic, and antioxidant properties of Santolina corsica Jord. & Fourr. (Asteraceae). Biomed. Pharmacother..

[13] Casaburi I., Avena P., De Luca A., Chimento A., Sirianni R., Malivindi R., Rago V., Fiorillo M., Domanico F., Campana C. (2015). Estrogen related receptor alpha (ERRalpha) a promising target for the therapy of adrenocortical carcinoma (ACC). Oncotarget.

[14] Li Y., Cappello A.R., Muto L., Martello E., Madeo M., Curcio R., Lunetti P., Raho S., Zaffino F., Frattaruolo L. (2018). Functional characterization of the partially purified Sac1p independent adenine nucleotide transport system (ANTS) from yeast endoplasmic reticulum. J. Biochem..

[15] Frattaruolo L., Carullo G., Brindisi M., Mazzotta S., Bellissimo L., Rago V., Curcio R., Dolce V., Aiello F., Cappello A.R. (2019). Antioxidant and Anti-Inflammatory Activities of Flavanones from Glycyrrhiza glabra L. (licorice) Leaf Phytocomplexes: Identification of Licoflavanone as a Modulator of NF-kB/MAPK Pathway. Antioxidants.

[16] Ozsvari B., Fiorillo M., Bonuccelli G., Cappello A.R., Frattaruolo L., Sotgia F., Trowbridge R., Foster R., Lisanti M.P. (2017). Mitoriboscins: Mitochondrial-based therapeutics targeting cancer stem cells (CSCs), bacteria and pathogenic yeast. Oncotarget.

[17] Dai X., Li T., Bai Z., Yang Y., Liu X., Zhan J., Shi B. (2015). Breast cancer intrinsic subtype classification, clinical use and future trends. Am. J. Cancer Res..

[18] Nicoletti I., Migliorati G., Pagliacci M.C., Grignani F., Riccardi C. (1991). A rapid and simple method for measuring thymocyte apoptosis by propidium iodide staining and flow cytometry. J. Immunol. Methods.

[19] Prieto A., Diaz D., Barcenilla H., Garcia-Suarez J., Reyes E., Monserrat J., San Antonio E., Melero D., de la Hera A., Orfao A. (2002). Apoptotic rate: A new indicator for the quantification of the incidence of apoptosis in cell cultures. Cytometry.

[20] Cai Z., Liu Q. (2017). Cell Cycle Regulation in Treatment of Breast Cancer. Adv. Exp. Med. Biol..

[21] Schafer K.A. (1998). The cell cycle: A review. Vet. Pathol..

[22] Giacinti C., Giordano A. (2006). RB and cell cycle progression. Oncogene.

[23] Seville L.L., Shah N., Westwell A.D., Chan W.C. (2005). Modulation of pRB/E2F functions in the regulation of cell cycle and in cancer. Curr. Cancer Drug Targets.

[24] Nagata S., Suzuki J., Segawa K., Fujii T. (2016). Exposure of phosphatidylserine on the cell surface. Cell Death Differ..

[25] Segawa K., Nagata S. (2015). An Apoptotic ‘Eat Me’ Signal: Phosphatidylserine Exposure. Trends Cell Biol..

[26] Kroemer G. (1999). Mitochondrial control of apoptosis: An overview. Biochem. Soc. Symp..

[27] Fulda S., Debatin K.M. (2006). Extrinsic versus intrinsic apoptosis pathways in anticancer chemotherapy. Oncogene.

[28] Armentano M.F., Bisaccia F., Miglionico R., Russo D., Nolfi N., Carmosino M., Andrade P.B., Valentao P., Diop M.S., Milella L. (2015). Antioxidant and proapoptotic activities of Sclerocarya birrea [(A. Rich.) Hochst.] methanolic root extract on the hepatocellular carcinoma cell line HepG2. Biomed Res. Int..

[29] Quijano C., Trujillo M., Castro L., Trostchansky A. (2016). Interplay between oxidant species and energy metabolism. Redox Biol..

[30] Johnson F., Giulivi C. (2005). Superoxide dismutases and their impact upon human health. Mol. Asp. Med..

[31] Kim Y.S., Gupta Vallur P., Phaeton R., Mythreye K., Hempel N. (2017). Insights into the Dichotomous Regulation of SOD2 in Cancer. Antioxidants.

[32] Rigoulet M., Yoboue E.D., Devin A. (2011). Mitochondrial ROS generation and its regulation: Mechanisms involved in H_2_O_2_ signaling. Antioxid. Redox Signal..

[33] De Luca A., Fiorillo M., Peiris-Pagès M., Ozsvari B., Smith D.L., Sanchez-Alvarez R., Martinez-Outschoorn U.E., Cappello A.R., Pezzi V., Lisanti M.P. (2015). Mitochondrial biogenesis is required for the anchorage-independent survival and propagation of stem-like cancer cells. Oncotarget.

[34] Berdy J. (2005). Bioactive microbial metabolites. J. Antibiot..

[35] Butler M.S., Robertson A.A., Cooper M.A. (2014). Natural product and natural product derived drugs in clinical trials. Nat. Prod. Rep..

[36] Spraker J.E., Luu G.T., Sanchez L.M. (2019). Imaging mass spectrometry for natural products discovery: A review of ionization methods. Nat. Prod. Rep..

[37] Bloudoff K., Schmeing T.M. (2017). Structural and functional aspects of the nonribosomal peptide synthetase condensation domain superfamily: Discovery, dissection and diversity. Biochim. Biophys. Acta Proteins Proteom..

[38] Shen B. (2003). Polyketide biosynthesis beyond the type I, II and III polyketide synthase paradigms. Curr. Opin. Chem. Biol..

[39] Sussmuth R.D., Mainz A. (2017). Nonribosomal Peptide Synthesis-Principles and Prospects. Angew. Chem..

[40] Lu J., Li J., Wu Y., Fang X., Zhu J., Wang H. (2019). Characterization of the FMN-Dependent Cysteine Decarboxylase from Thioviridamide Biosynthesis. Org. Lett..

[41] Mahanta N., Liu A., Dong S., Nair S.K., Mitchell D.A. (2018). Enzymatic reconstitution of ribosomal peptide backbone thioamidation. Proc. Natl. Acad. Sci. USA.

[42] Haque R., Ahmed S.A., Inzhakova G., Shi J., Avila C., Polikoff J., Bernstein L., Enger S.M., Press M.F. (2012). Impact of breast cancer subtypes and treatment on survival: An analysis spanning two decades. Cancer Epidemiol. Prev. Biomark..

[43] Yersal O., Barutca S. (2014). Biological subtypes of breast cancer: Prognostic and therapeutic implications. World J. Clin. Oncol..

[44] Massague J. (2004). G1 cell-cycle control and cancer. Nature.

[45] Singh A., Kukreti R., Saso L., Kukreti S. (2019). Oxidative Stress: A Key Modulator in Neurodegenerative Diseases. Molecules.

[46] Phan L.M., Yeung S.C., Lee M.H. (2014). Cancer metabolic reprogramming: Importance, main features, and potentials for precise targeted anti-cancer therapies. Cancer Biol. Med..

[47] Seyfried T.N., Shelton L.M. (2010). Cancer as a metabolic disease. Nutr. Metab..

[48] Pelicano H., Martin D.S., Xu R.H., Huang P. (2006). Glycolysis inhibition for anticancer treatment. Oncogene.

[49] Guo Z., Sevrioukova I.F., Denisov I.G., Zhang X., Chiu T.L., Thomas D.G., Hanse E.A., Cuellar R.A.D., Grinkova Y.V., Langenfeld V.W. (2017). Heme Binding Biguanides Target Cytochrome P450-Dependent Cancer Cell Mitochondria. Cell Chem. Biol..

[50] Armstrong J.A., Cash N.J., Ouyang Y., Morton J.C., Chvanov M., Latawiec D., Awais M., Tepikin A.V., Sutton R., Criddle D.N. (2018). Oxidative stress alters mitochondrial bioenergetics and modifies pancreatic cell death independently of cyclophilin D, resulting in an apoptosis-to-necrosis shift. J. Biol. Chem..

[51] Chae Y.C., Kim J.H. (2018). Cancer stem cell metabolism: Target for cancer therapy. BMB Rep..

[52] Snyder V., Reed-Newman T.C., Arnold L., Thomas S.M., Anant S. (2018). Cancer Stem Cell Metabolism and Potential Therapeutic Targets. Front. Oncol..

[53] Vlashi E., Pajonk F. (2015). The metabolic state of cancer stem cells-a valid target for cancer therapy?. Free Radic. Biol. Med..

