# Supplementary material for: Thioalbamide, A Thioamidated Peptide from Amycolatopsis alba, Affects Tumor Growth and Stemness by Inducing Metabolic Dysfunction and Oxidative Stress
Source: Cells. 2019 Nov 8;8(11):1408. doi: 10.3390/cells8111408 (PMC6912574; doi:10.3390/cells8111408)
Supplement: Supplementary file 1 [file cells-08-01408-s001.pdf]

cells

MDPI

SUPPLEMENTARY FIGURES

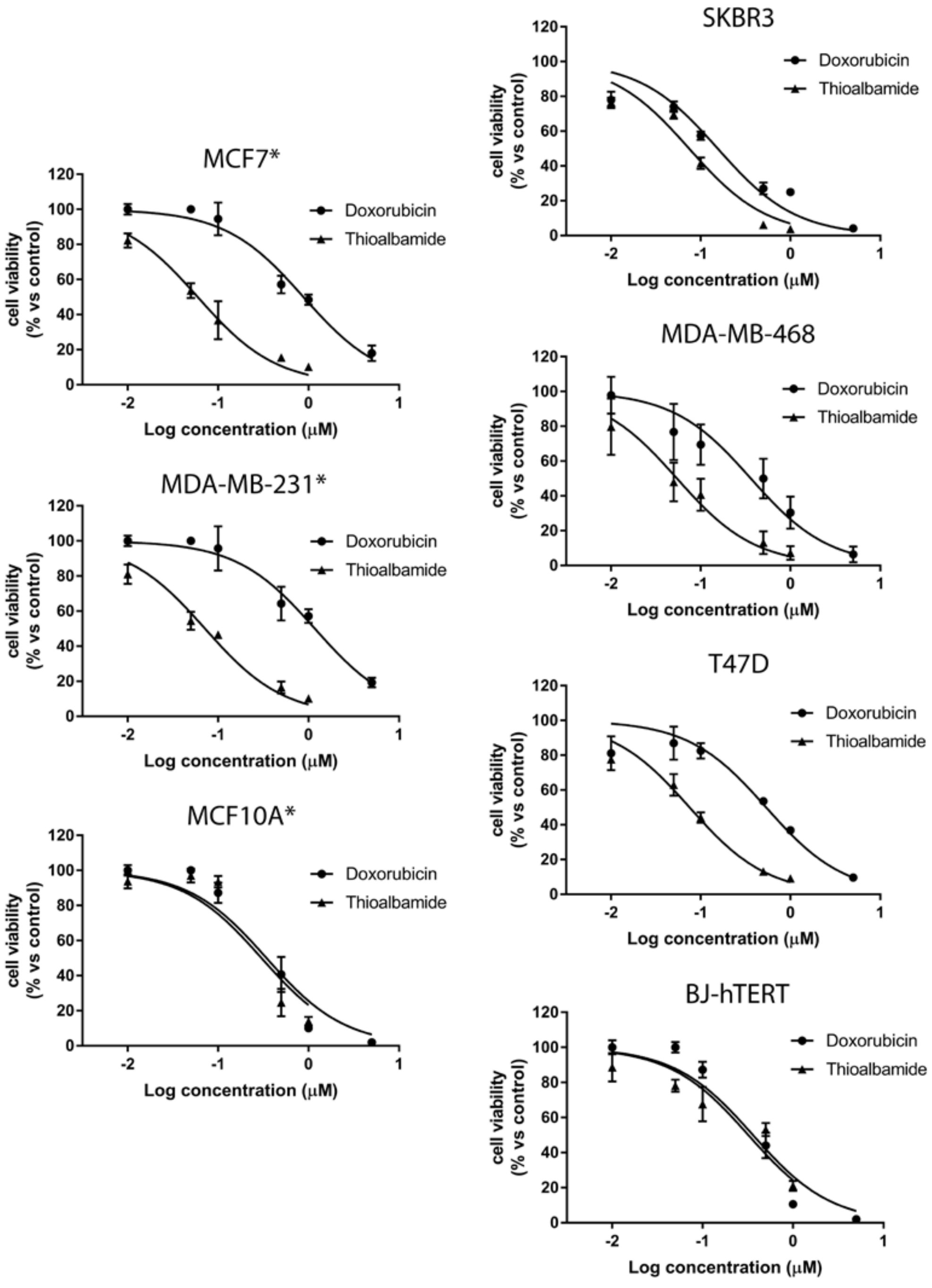

Figure S1. Thioalbamide affects breast cancer cellular growth.
Cellular growth assessment after treatment of MCF7, MDA-MB-231, MDA-MB-468, T47D, SKBR3, MCF-10A and BJ-hTERT cell lines with different concentrations of thioalbamide (0.01 to 1 μM) or Doxorubicin (0.01 to 5 μM) for 72 h. Results, quantified by the MTT assay, are expressed as percentage of growth vs control cells treated with vehicle alone (DMSO). Values represent mean ± SD of three independent experiments, each performed with triplicate samples.
*Results reported in Frattaruolo et al. 2017.

cells

MDPI

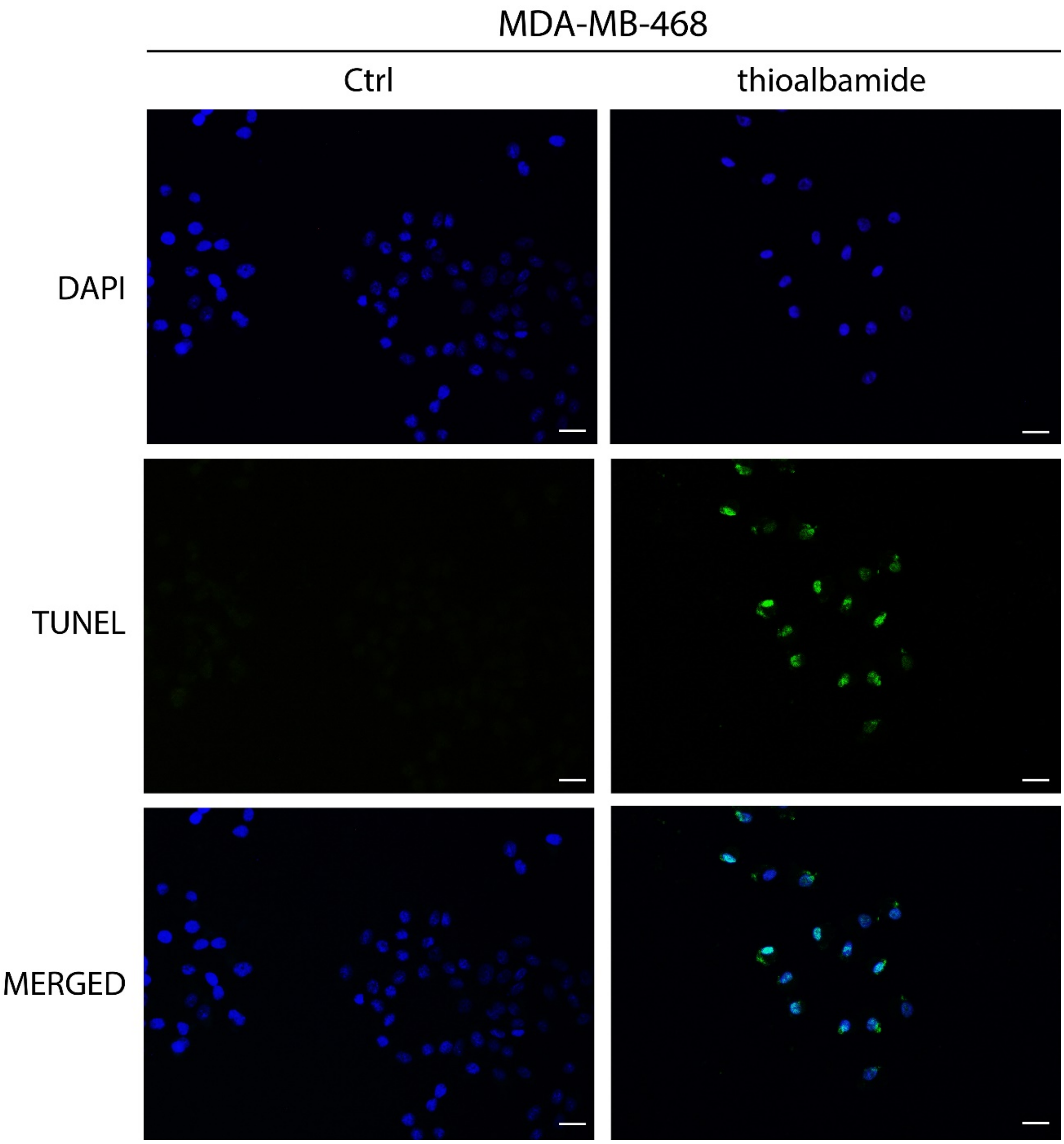

Figure S2. Thioalbamide-induced DNA fragmentation in MDA-MB-468 cells. TdT-mediated dUTP nick-end-labeling (TUNEL) assay in MDA-MB-468 cells treated for 72 h with vehicle (Ctrl) or 50 nM thioalbamide. DAPI was used for DNA staining. Images at 20X magnification were taken with an Olympus BX41 microscope with CSV1.14 software, using a CAMXC-30 for image acquisition (scale bar: 25 μm)

cells

MDPI

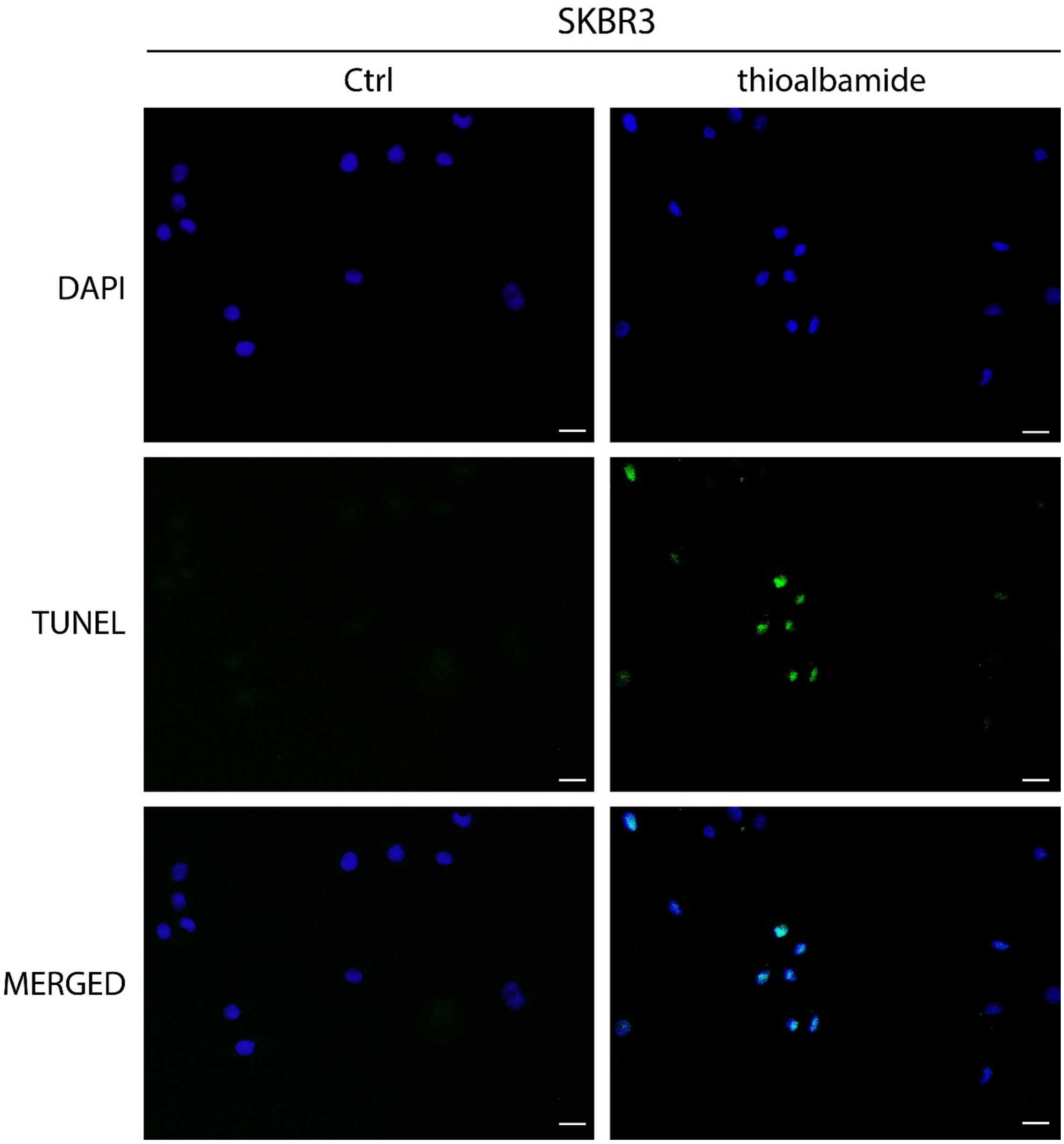

Figure S3. Thioalbamide-induced DNA fragmentation in SKBR3 cells. TdT-mediated dUTP nick-end-labeling (TUNEL) assay in SKBR3 cells treated for 72 h with vehicle (Ctrl) or 100 nM thioalbamide. DAPI was used for DNA staining. Images at 20X magnification were taken with an Olympus BX41 microscope with CSV1.14 software, using a CAMXC-30 for image acquisition (scale bar: 25 μm)

cells MDPI

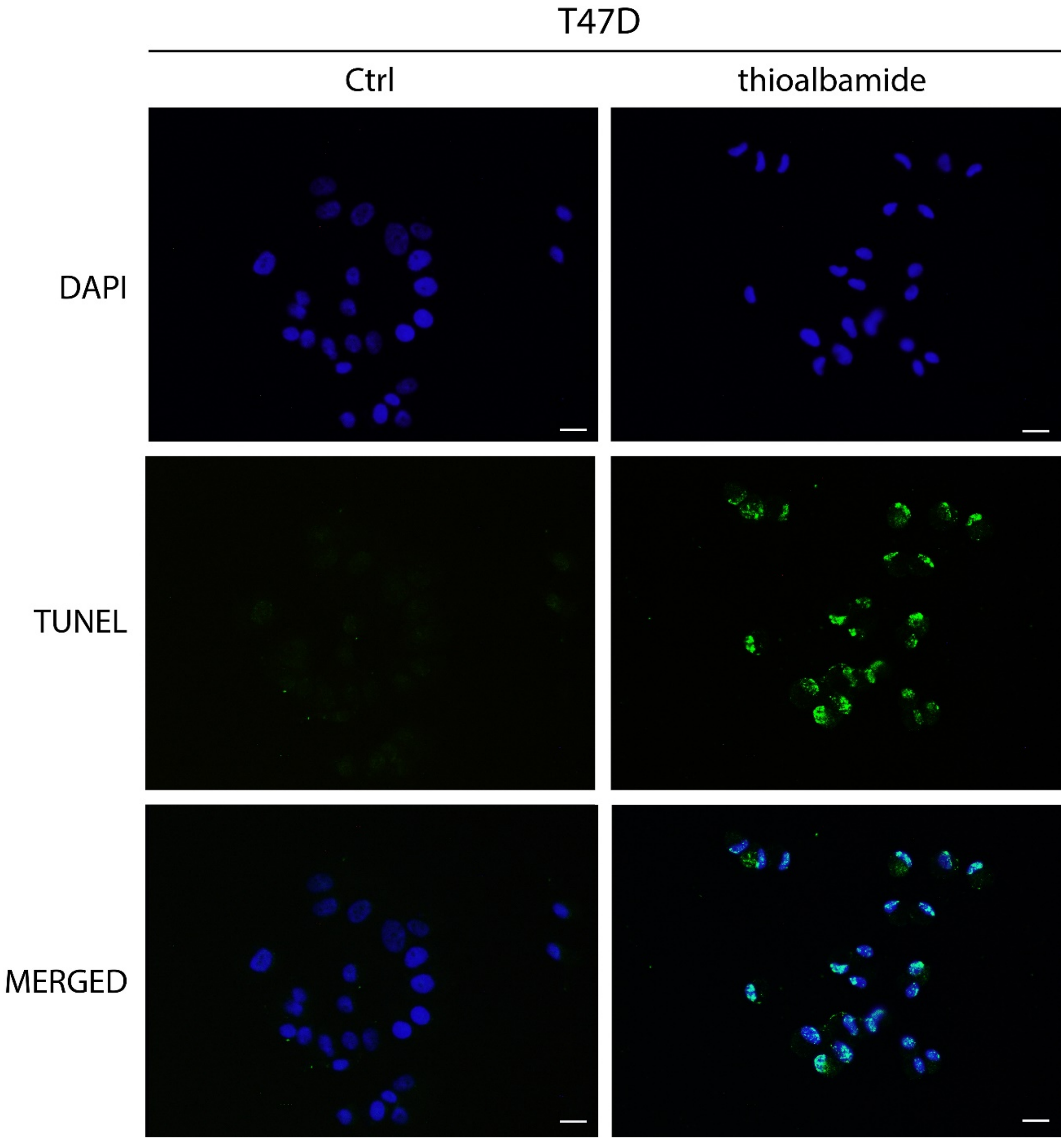

Figure S4. Thioalbamide-induced DNA fragmentation in T47D cells. TdT-mediated dUTP nick-end-labeling (TUNEL) assay in T47D cells treated for 72 h with vehicle (Ctrl) or 50 nM thioalbamide. DAPI was used for DNA staining. Images at 20X magnification were taken with an Olympus BX41 microscope with CSV1.14 software, using a CAMXC-30 for image acquisition (scale bar: 25 µm)

cells

MDPI

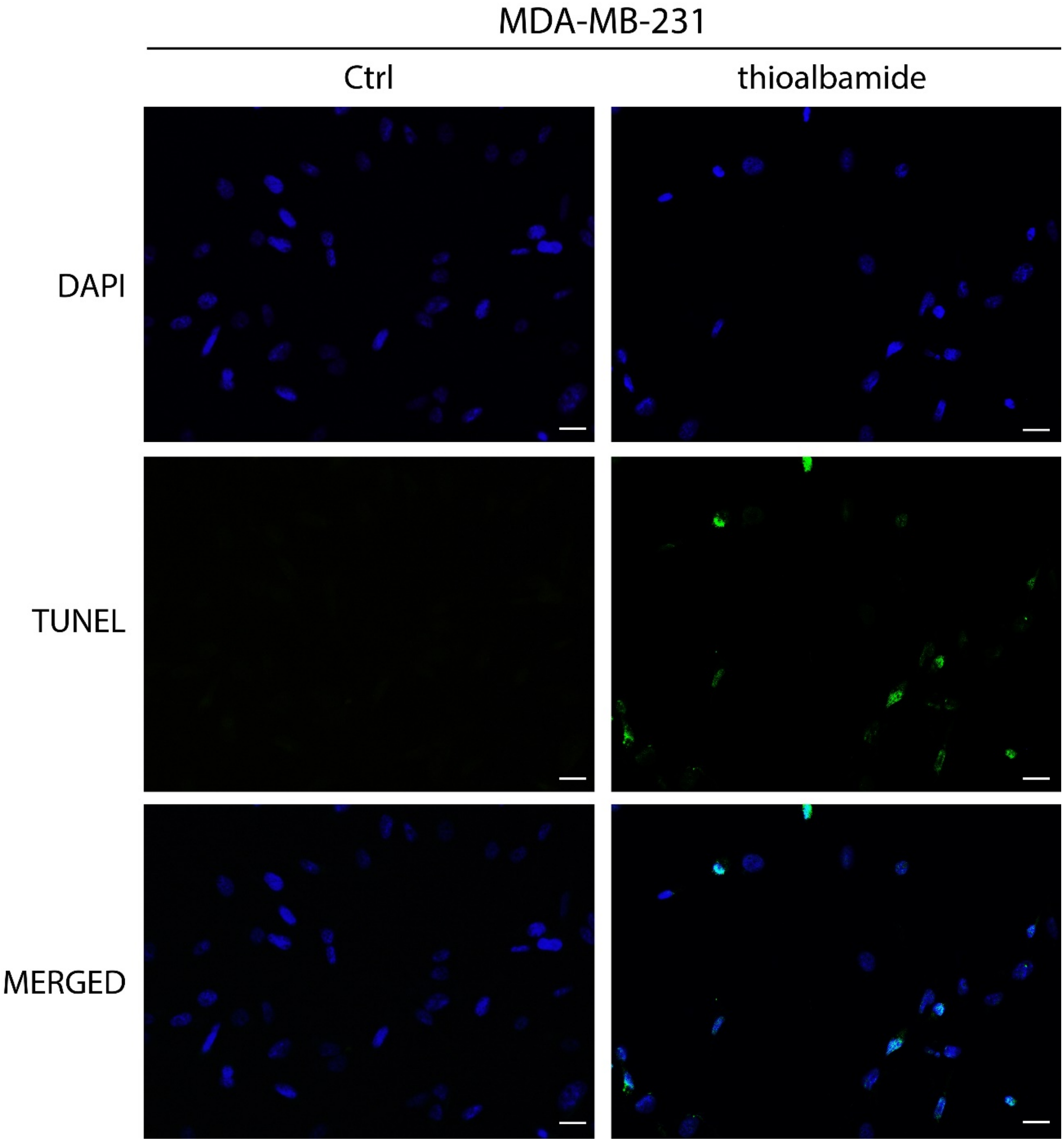

Figure S5. Thioalbamide-induced DNA fragmentation in MDA-MB-231 cells. TdT-mediated dUTP nick-end-labeling (TUNEL) assay in MDA-MB-231 cells treated for 72 h with vehicle (Ctrl) or 100 nM thioalbamide. DAPI was used for DNA staining. Images at 20X magnification were taken with an Olympus BX41 microscope with CSV1.14 software, using a CAMXC-30 for image acquisition (scale bar: 25 µm)

cells
MDPI

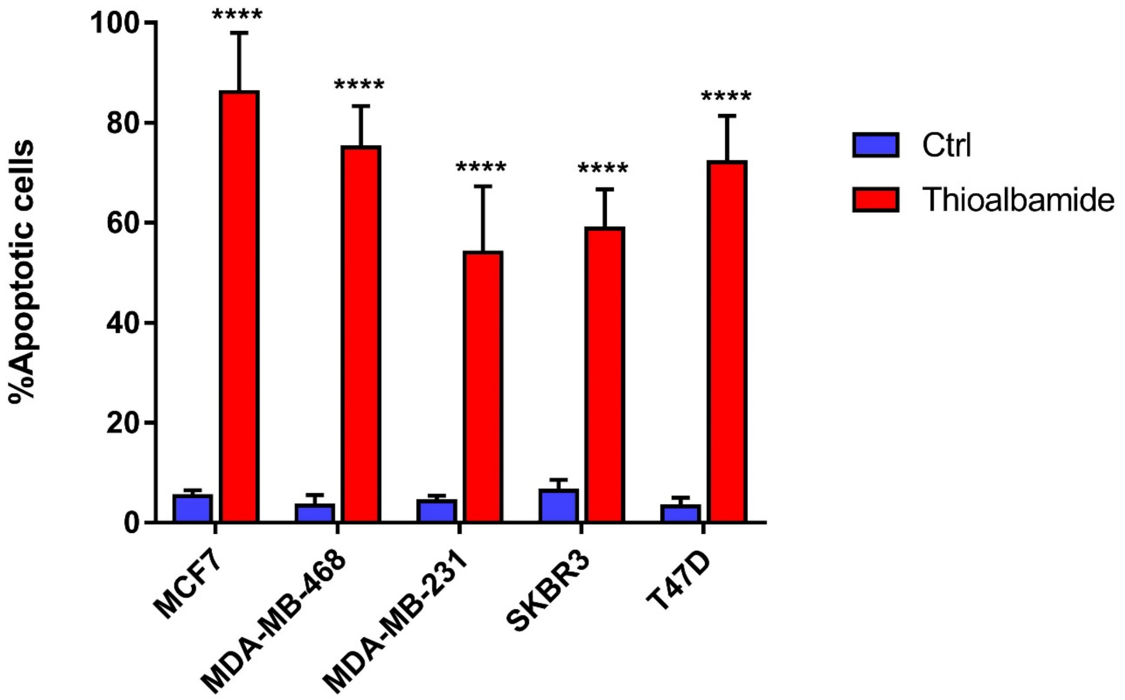

Figure S6. Thioalbamide induces apoptosis in breast cancer cell lines. Quantification of positive cells in TUNEL assay performed on MCF7, MDA-MB-468, MDA-MB-231, SKBR3 and T47D. Values represent mean ± SD of three independent experiments. ****P value <0.0001

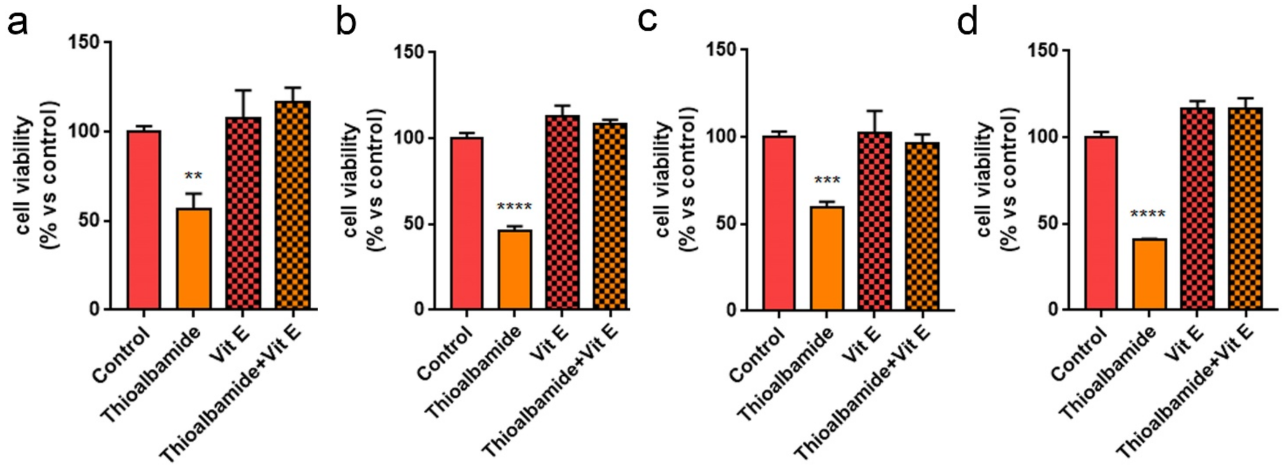

Figure S7. Oxidative stress underlies thioalbamide cytotoxicity in breast cancer cell lines. Cell viability assessment of MDA-MB231 (a), MDA-MB-468 (b), T47D (c) and SKBR3 (d) cells after treatment for 72 h with 50 nM thioalbamide, alone or in association with vitamin E (Vit E), as indicated. **P value <0.01; ***P value <0.001; ****P value <0.0001

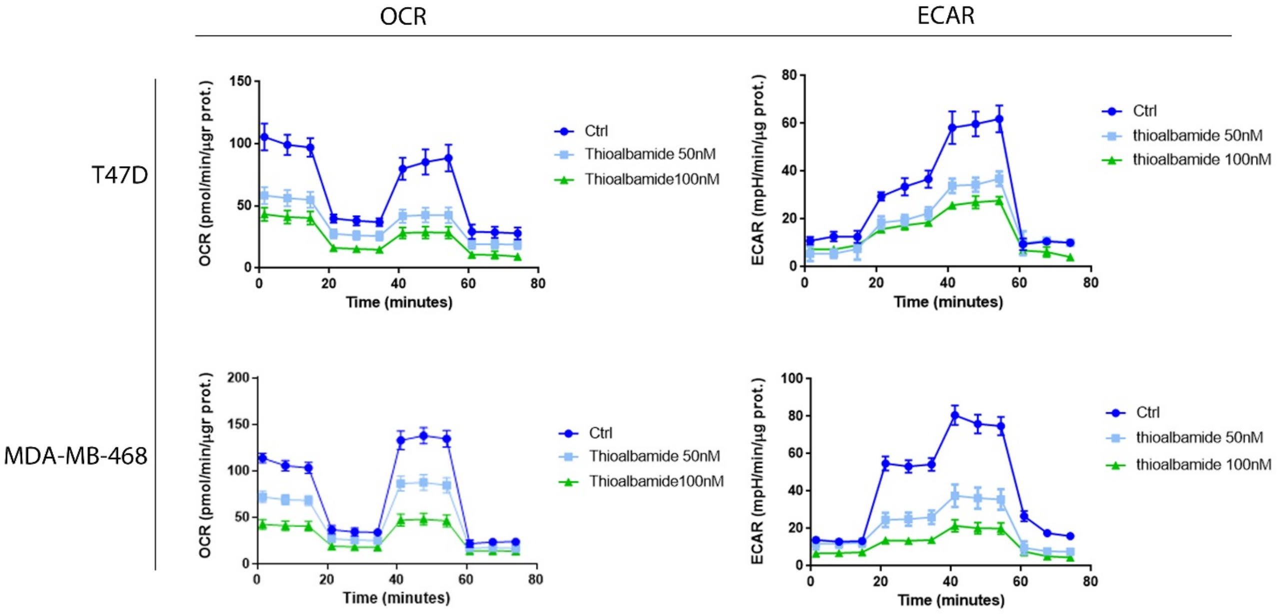

Figure S8. Metabolic profile of breast cancer cells treated with thioalbamide. Metabolic profile (OCR and ECAR) of T47D and MDA-MB-468 cells treated with 50/100 nM thioalbamide for 48 h.Values represent mean ± SEM of three indipendent experiments

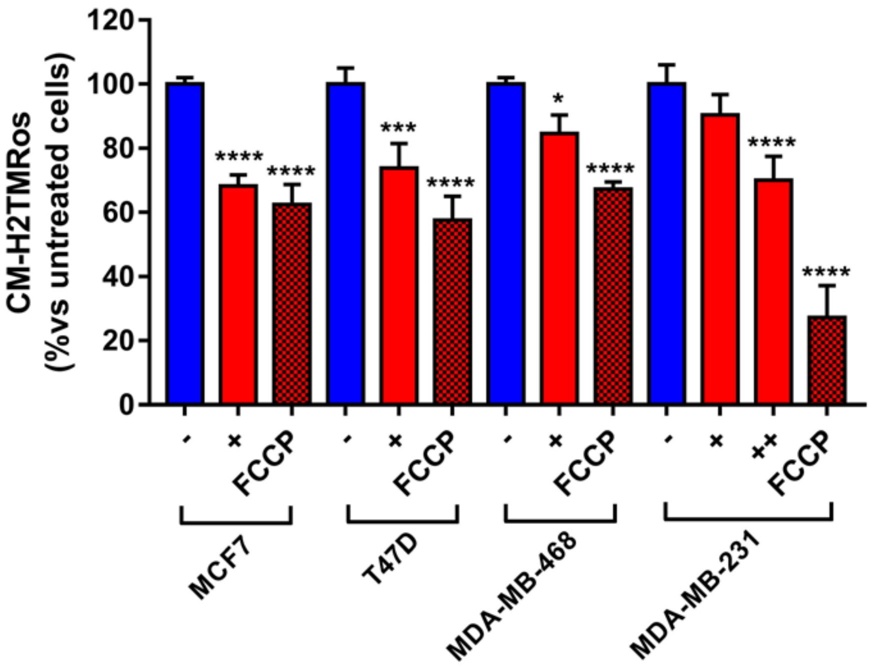

Figure S9. Thioalbamide induces loss of mitochondrial membrane potential in breast cancer cells. Mitochondrial membrane potential was assessed after 72 h of treatment with thioalbamide, using MitoTracker Orange CM-H2TMRos probe. 50.000 events were acquired using SONY SH800 flow cytometer. Values represent mean ± SD of three independent experiments. Treatment conditions: DMSO (-), 50 nM thioalbamide (+), 100 nM thioalbamide (++). 2µM FCCP was used as positive control. *P value <0.05; ****P value <0.0001

cells

MDPI

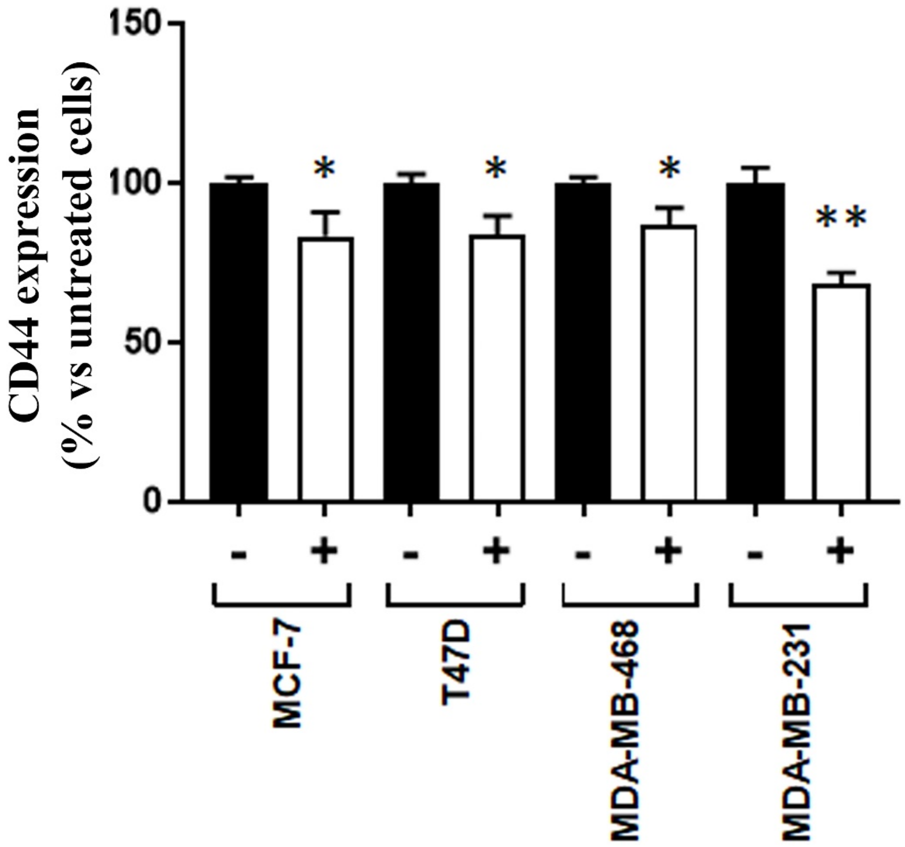

Figure S10. Thioalbamide reduces CD44 expression in breast cancer cells. CD44 antibody staining was assessed, after 72h of treatment with thioalbamide, using anti-human CD44 (BD Bioscience). 50.000 events were acquired using SONY SH800 flow cytometer. Values represent mean ± SD of three independent experiments. Treatment conditions: DMSO (-), 50 nM thioalbamide (+). *P value <0.05; **P value <0.01
